# Synuclein-γ suppression mediated by RNA interference inhibits the clonogenicity and invasiveness of MCF-7 cells

**DOI:** 10.3892/ol.2013.1192

**Published:** 2013-02-14

**Authors:** BO LIANG, XIN-JUN WANG, PEI-HONG SHEN, XUE-YUAN LI, HONG-WEI CHENG, QIAO SHAN, KUI-YUAN GUO, YU-WEN CAO, QING-XIA FAN, RUI-FENG ZHENG, BEI LI, WEI ZHANG, YAN-WEI LI, KAI YANG

**Affiliations:** 1 Departments of Neurosurgery, Zhengzhou, Henan 450052, P.R. China;; 2 Pathology, The Fifth Affiliated Hospital, Zhengzhou, Henan 450052, P.R. China;; 3 General Surgery, The Fifth Affiliated Hospital, Zhengzhou University, Zhengzhou, Henan 450052, P.R. China;; 4 Department of Experimental Therapeutics, BC Cancer Agency Research Centre, Vancouver, British Columbia V5Z1L3, Canada;; 5 Department of Oncology, The First Affiliated Hospital, Zhengzhou University, Zhengzhou, Henan 450051;; 6 Center of Cancer Therapy, Henan People’s Armed Police Corps Hospital, Zhengzhou, Henan 450052, P.R. China

**Keywords:** breast cancer, MCF-7 cell line, RNA interference, synuclein-γ

## Abstract

The aim of the present study was to investigate the effects of synuclein-γ (SNCG) downregulation by RNA interference (RNAi) on the clonogenicity and invasiveness of MCF-7 breast cancer cells. This study used four pairs of SNCG-specific siRNAs which were designed and cloned into the pGPU6 plasmid for introduction into an MCF-7 cell line. The SNCG knockdown efficacies of the four siRNAs were compared using the reverse transcription polymerase chain reaction (RT-PCR) and immunocytochemistry. The cells’ clonogenic and invasive phenotypes were examined with clonogenic and Boyden chamber assays. In comparison with the non-specific siRNA and empty vector controls, all four SNCG siRNAs were observed to significantly inhibit SNCG expression at the mRNA and protein levels (F=481.06, P<0.001; F=147.42, P<0.0001). SNCG suppression mediated by RNAi successfully inhibited the clonogenicity (P=0.002) and invasiveness (P<0.001) of transfected MCF-7 cells. According to the results of the present study, we concluded that SNCG suppression mediated by RNAi significantly suppressed SNCG expression at the mRNA and protein levels, suggesting that SNCG suppression mediated by an RNAi strategy may become a novel approach for treating advanced breast cancer.

## Introduction

Breast cancer is the most commonly occurring malignancy in women. Worldwide, it is estimated that more than one million women are diagnosed with breast cancer each year and >410,000 succumb to the disease (1). Despite great improvements over the past decades in conventional breast cancer treatment approaches, including surgery, radiotherapy, chemotherapy and endocrine therapy, breast cancer recurrence, metastasis and mortality rates remain high. With the considerable progress in molecular cancer biology, targeted therapy is emerging as the most promising strategy in cancer therapeutics. In breast cancer, the use of trastuzumab treatment in Her-2-positive patients has been considered as a successful example of modern oncology (2). However, given the complexity of breast cancer biology itself and the limited application range of each targeted therapy, the exploration of new cancer targets with the aim of developing novel therapeutic approaches remains a pressing need in breast cancer research.

Synuclein-γ (SNCG) is a human gene localized at 10q23.20–23.3. SNCG cDNA is ∼5 kb in length and comprised of five exons that translate into a protein of 127 amino acids (3,4). The SNCG protein belongs to the family of synuclein proteins (5). Synucleins are small soluble proteins expressed primarily in the neural tissue and certain tumors (6). The three synucleins that comprise the entire protein family (α-synuclein, β-synuclein and SNCG) are characterized by a highly conserved α-helical lipid-binding motif with similarity to the class-A2 lipid-binding domains of the exchangeable apolipoproteins (7). Members of this family are considered to have important pathogenic roles in various neurodegenerative disorders, including Parkinson’s disease, dementia with Lewy bodies and Alzheimer’s disease (8–10). Since SNCG was first observed to be overexpressed in advanced breast carcinoma (11), but not in normal or benign breast tissue, it was previously named breast cancer specific gene 1 (BCSG1). At present, accumulating evidence has shown that the over-expression of this gene is also implicated in numerous other cancer types, including ovarian, lung, liver, esophagus, colon and prostate (12–15), and correlated with the later stages of the diseases. In addition, SNCG was also suggested to be associated with antimicrotubule drug resistance in breast cancer treatment (16). Gene function studies further indicated that SNCG is associated with tumor progression through a series of mechanisms which include the stimulation or promotion of cell proliferation, invasion and metastasis (17–20). All this evidence indicated that SNCG is a tumor marker and a knockdown of SNCG oncogene expression may be an effective strategy in breast cancer treatment.

RNA interference (RNAi) is a specific gene silencing phenomenon mediated by double-stranded RNA, which is an ancient and highly conserved gene regulatory mechanism (21). Since its discovery, RNAi technology has rapidly become an effective gene-silencing tool and has been broadly applied in a number of biological research fields. The double-stranded small RNAi molecule functions in the nanomolar range and is far more effective than the antisense approach which was popular 10–15 years ago. In cancer research, RNAi intervention has been used for targeting numerous gene products involved in carcinogenesis, including key molecules crucial for cancer cell proliferation, tumor-host interactions and tumor resistance to chemo- or radiotherapy. With the ever-evolving understanding of molecular cancer biology, great opportunities for using RNAi technology in cancer therapy are rapidly being created. The selection of appropriate gene targets is also becoming a critical parameter for the potential success of RNAi-based cancer therapies.

In the present study, a vector-based RNAi strategy was used to suppress the expression of SNCG in the breast cancer MCF-7 cell line and its effects on the clonogenicity and invasion of the transfected cells were evaluated. It was observed that the specific downregulation of SNCG was achieved at the mRNA and protein levels by this approach and the clonogenic and invasion capabilities of the cancer cells were significantly diminished. We concluded that SNCG suppression mediated by RNAi may become an effective therapeutic approach for the treatment of breast cancer.

## Materials and methods

### Cell culture

The human breast cancer MCF-7 cells were purchased from Nanjing Kenou Biology Co. Ltd. (Nanjing, China) and grown in RPMI-1640 culture medium containing 10% fetal bovine serum (FBS), 100 U/ml penicillin and 100 U/ml streptomycin in 75 cm^2^ flasks and incubated in a humidified incubator (37°C, 5% CO_2_). The study was approved by the Institutional Review Board of Zhengzhou University, Zhengzhou, China.

### Construction of SNCG RNAi plasmid expression vectors

Based on the full SNCG cDNA sequence, four pairs of siRNA DNA chains were designed using Ambion and Takara siRNA design programs to produce the following sequences: 5′-CCAA GGAGAATGTTGTACAGA-3′, 5′-CAAGACCAAGGAGAAT GTTGT-3′, 5′-GCCAAGACCAAGGAGAATGTT-3′ and 5′-TGGTGAGCAGCGTCAACACTG-3′. The negative control siRNA was designed as 5′-GTTCTCCGAACGTGTCAC GT-3′, a sequence with no homology to human or mouse SNCG. All designed sequences were then synthesized and constructed into pGPU6 vector (Shanghai GenePharma Co. Ltd, Shanghai, China).

### Transfection of plasmid DNA and selection of stable cell lines

MCF-7 cells were seeded in a 24-well plate one day prior to transfection and transfected with 1.6 *μ*g SNCG RNAi plasmid DNA and 4 *μ*l transfection reagent Lipofectamine 2000 (Invitrogen Life Technologies, Carlsbad, CA, USA) when the cells reached ∼90% confluence. Together with the four SNCG specific RNAi plasmids, transfection of a non-specific RNAi negative control plasmid, an empty vector control plasmid and the Lipofectamine 2000 control were also performed. After 48 h, the transfected cells were plated on to 6-well plates and selected with G418 at 800 mg/l. Fresh culture media containing G418 was replaced every two or three days and stable cell lines were established in ∼2 weeks. The RNAi expression status in all stable lines was visually monitored under microscope by GFP signal.

### Immunocytochemistry assay

The SNCG expression level was detected using the SP hypersensitivity kit (Beijing Biosynthesis Biotechnology Co., Ltd., Beijing, China) according to the manufacturer’s instructions. SNCG primary antibody was purchased from Santa Cruz Biotechnology Inc. (Santa Cruz, CA, USA) and PBS was applied as negative control. Positive SNCG staining was shown as yellow to brown signals located in the cytoplasm. Under a light microscope, 10 high power fields were randomly selected and scored with the following criteria (22): a) for the extent of positive staining: 0 = positive cells <1%, 1 = 1–25%, 2 = 26–50%, 3 = 51–75% and 4 = 76–100%; b) for staining intensity: 0 = no color, 1 = pale-yellow, 2 = brown to yellow, 3 = tan. The final score was equal to a × b and was used for statistical analysis.

### Reverse transcription polymerase chain reaction (RT-PCR) assay

Cells (5×10^5^) from each SNCG RNAi-stable cell line and negative controls were used for total RNA extraction using the TRIzol reagent (Invitrogen Life Technologies). The RNA concentration and quality were determined with an ultraviolet spectrophotometer. The reverse transcription reaction was performed using the RT-PCR kit purchased from Invitrogen Life Technologies. The primer sequences used for SNCG detection were as follows: upstream primer, 5′-CAAGAAGGG CTTCTCCATCGCCAAGG-3′ and downstream primer, 5′-CCTCTTTCTCTTTGGATGCCACACCC-3′. *ACTB* was used as the internal control and the primer sequences were: upstream primer, 5′-GATGACGATATCGCTGCGCT-3′ and downstream primer, 5′-CTAGGCACCAGGTAAGTGAC-3′. The PCR amplification reaction was as follows: 94°C for 30 sec, 55°C for 30 sec, 72°C for 1 min (for 30 cycles) and a final extension at 72°C for 10 min. The PCR products were analyzed by electrophoresis on a 1% agarose gel and were visualized by ethidium bromide staining under ultraviolet light. The expression level of SNCG was indicated by the photodensity of the PCR band on the image and normalized with the corresponding internal ACTB band intensity for each sample.

### Clonogenic assay

Along with parallel MCF-7 cells, empty vector and non-specific transfection cells, SNCG-RNAi-3-stable cells (with the most potent SNCG knockdown efficiency) were grown in RPMI-1640 media (with 10% FBS) to 70% confluence, trypsinized and counted. Cells (2×10^4^) were seeded on 60-mm tissue culture dishes (in triplicate) and incubated for 14 days. Fresh media were added every three to five days. At day 14, the culture media were removed and the cells were washed with PBS three times. Cells were fixed with methanol for 15 min at room temperature, followed by hemotoxylin staining for 2–3 min and washed with PBS. All colonies with a cell count >50 were identified under a microscope. The clonogenic rate was calculated as: average colony number / seeded cell number × 100.

### Boyden chamber cell invasiveness assay

The cell invasion assay was performed using Boyden chambers (Corning Co., Corning, NY, USA). Cells (5×10^5^) from each stable and control cell line were suspended in 100 *μ*l serum-free medium and placed into the upper compartments of the Boyden chambers. The lower compartments of the chambers were filled with 200 *μ*l serum-containing medium and the cells were allowed to migrate for 24 h. Following the incubation, the cells on the lower surface of the filter were fixed in cold ethanol and stained with 0.5% crystal violet (CV) for 30 min. Five random fields were then selected and counted at magnification ×200. Data representing the average cell numbers of the five fields were compared between all the experimental and control groups.

### Statistical analysis

All results were expressed as the mean ± SD. Statistical analyses were performed using SPSS 10.0 statistical software to perform t-tests. All statistical analyses were two-sided and comparisons were made in which P<0.05 was considered to indicate statistically significant differences.

## Results

### SNCG RNAi significantly inhibits SNCG mRNA expression

**Figure 1:**
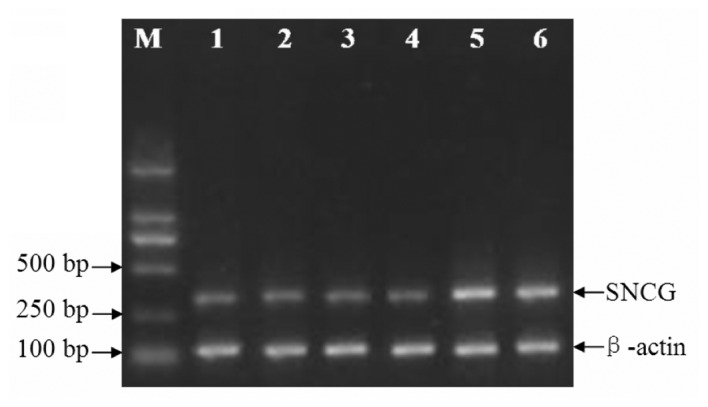
Downregulation of synuclein-γ (SNCG) detected by reverse transcription polymerase chain reaction. Lanes 1–4, SNCG-siRNA 1–4; lane 5, non-specific siRNA; lane 6, empty vector control.

Four SNCG RNAi expression plasmids (SNCG-siRNA-1, -2, -3 and -4) and a non-specific plasmid were constructed in pGPU-6 vectors. After the corresponding stable cell lines were established, the SNCG mRNA expression levels were measured with RT-PCR and compared among the groups. As demonstrated in Fig. 1 and Table I, SNCG mRNA expression was significantly diminished in the four SNCG RNAi groups in comparison with the parallel control (P<0.05), whereas the levels stayed almost unchanged in the non-specific RNAi and empty vector groups (P>0.05).

### SNCG RNAi significantly inhibits SNCG protein expression

**Figure 2:**
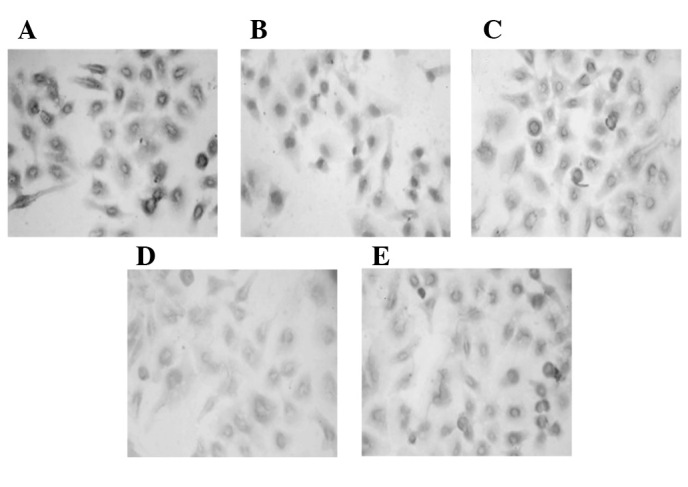
Immunohistochemical detection of synuclein-γ (SNCG) expression in breast cancer MCF-7 cells transfected with SNCG-siRNA. (A) Empty vector control; (B) SNCG-siRNA-1; (C) SNCG-siRNA-2; (D) SNCG-siRNA-3); (E) SNCG-siRNA-4. Streptavidin-peroxidase (SP), ×200.

The SNCG protein expression levels were assessed by immunocytochemistry for all the stable cell lines, followed by statistical analyses. In accordance with the results for SNCG expression at the mRNA level, SNCG protein expression was also significantly reduced in the four SNCG RNAi groups (P<0.05), in comparison with the parallel controls (Fig. 2 and Table II).

### SNCG suppression mediated by RNAi significantly diminishes the clonogenicity of transfected MCF-7 cells

**Figure 3:**
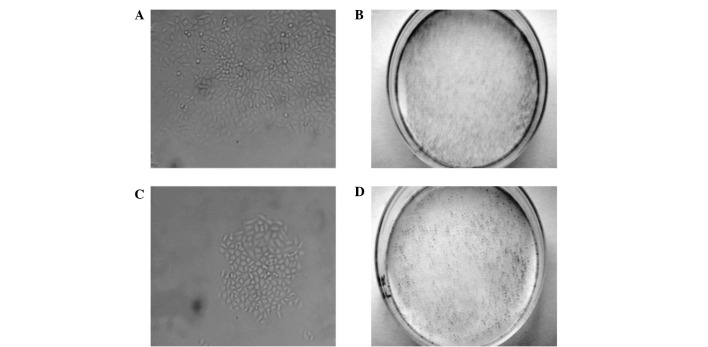
Effects of SNCG-siRNA suppression on the clonogenicity of MCF-7 cells. The colonies formed in (C,D) the SNCG-siRNA-3 line were smaller and fewer than those of (A,B) the parallel control. SNCG, synuclein-γ.

To investigate the effects of SNCG suppression mediated by RNAi on the clonogenicity of MCF-7 cells, the SNCG-siRNA-3-stable cell line (with the most potent SNCG knock-down efficiency) was used for comparison with the parallel and other transfection controls. As demonstrated in Figs. 3 and 5A, the sizes and number of colonies formed in the SNCG-siRNA-3 line were observed to be smaller and lower than in the parallel control, respectively. The clonogenic rate was significantly reduced (P<0.05) in comparison with the controls (Table III), whereas no significant difference was observed in the non-specific and empty vector controls compared with the parallel control.

### SNCG suppression mediated by RNAi significantly inhibits the invasiveness of transfected MCF-7 cells

**Figure 4:**
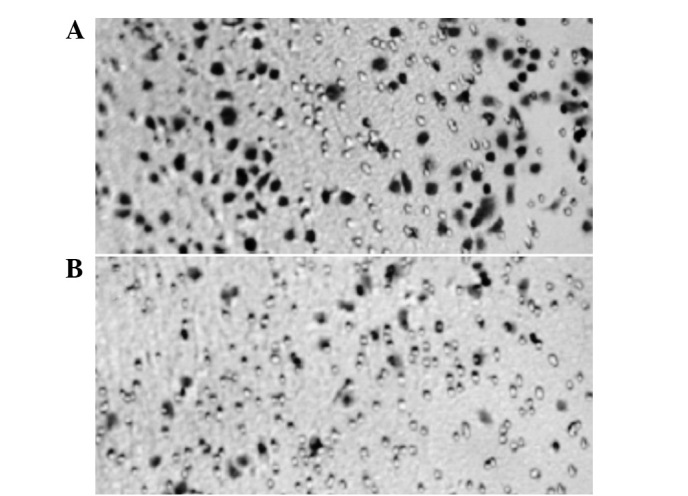
Effects of SNCG-siRNA suppression on the invasiveness of MCF-7 cells. The number of migrated cells in (B) SNCG-siRNA-3 line was much lower than that in (A) the parallel control. SNCG, synuclein-γ.

**Figure 5:**
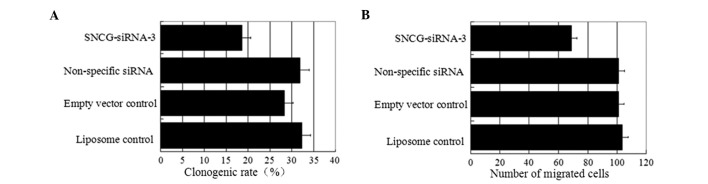
Differences in the clonogenic rate and number of migrated cells between the test group and various control groups. (A) The clonogenic rate was significantly reduced in SNCG-siRNA-3 line, whereas no significant differences were observed among the parallel controls. (B) The number of migrated cells was observed to be lower in the SNCG-siRNA-3 line compared with the parallel controls (B). SNCG, synuclein-γ.

The effects of SNCG suppression mediated by RNAi on cell invasiveness were assessed by Boyden chamber assays. As shown in Figs. 4 and 5B, the number of migrated cells was observed to be lower in the SNCG-siRNA-3 line compared with the parallel control. The differences in invasiveness between SNCG-siRNA-3 and all other controls were significant, as demonstrated in Table IV, whereas no significant differences were observed among the controls.

## Discussion

SNCG has been shown to be an unfavorable prognostic marker in a variety of cancer types (11–15). In breast cancer, the expression of SNCG was observed to be closely correlated with the disease stage, lymph node involvement, metastasis, tumor size and Her-2 status, but not with estrogen receptor (ER) and progesterone (PR) expression status (23). Overexpression of SNCG in breast cancer cells leads to the significant stimulation of cell proliferation (19), increased invasiveness and profound augmentation of metastasis in nude mice (24). Moreover, SNCG was also reported to be correlated with ERα overexpression (20), antimicrotubule drug resistance (25) and an accelerated rate of chromosomal instability (26). All these findings suggest that SNCG may be a potential therapeutic target in breast cancer treatment.

Several strategies using growth factor inhibitor and antagonistic peptides for targeting aberrant SNCG expression have been investigated (27,28). Although they have been reported as having a level of effectiveness, the clinical application of these approaches remains far away. Given that RNAi is such a well-developed technology and effective delivery systems for RNAi are under intensive investigation, we proposed that it would be rational to study an SNCG targeting strategy mediated by RNAi instead of other systems. In the present study, a series of SNCG siRNA sequences were designed and validated, constructed into siRNA expression vectors and introduced into MCF-7 cells. The present results show that all these small molecules were able to specifically and significantly suppress the SNCG expression at the mRNA and protein levels. At the maximum response, 35.81 and 48.25% reductions in SNCG mRNA and protein expression, respectively, were observed in stable SNCG RNAi lines. By contrast, SNCG expression was almost unchanged in the control groups.

On the basis of the significant suppressive effects on SNCG expression, the effects of these SNCG RNAi molecules on the clonogenicity and invasiveness of breast cancer cells were further investigated. In comparison with the parallel MCF-7 control, as much as 50.82% inhibition in clonogenicity and 44.33% in invasiveness was observed in SNCG RNAi-transfected MCF-7 cells, indicating that successful SNCG downregulation was able to attenuate breast cancer malignancy and may possibly be a novel approach for cancer treatment.

The mechanisms underlying SNCG’s involvement in breast cancer pathogenesis have been studied in previous studies. In addition to its involvement in the checkpoint complex (26), SNCG has also been shown to stimulate the ligand-dependent transcriptional activity of ER in breast cancer cells (20). It has also been shown that SNCG stimulates ER signaling by acting as a chaperone through a multi-protein chaperone complex with Hsp70 and Hsp90 (19). Given the essential role of estrogen in modulating the cellular growth and differentiation of the mammary gland, any factors affecting this signaling pathway should be considered to be critical in breast cancer pathogenesis. Moreover, the overexpression of SNCG was demonstrated to correlate with elevated levels of matrix metalloproteases (MMPs) (20,24,29), a well recognized mechanism associated with the characteristic invasive and metastatic properties of cancer cells (29).

Taken together, the present findings that SNCG suppression mediated by RNAi may significantly diminish breast cancer cell malignancy in terms of clonogenicity and invasiveness, suggested that SNCG may be exploited as a therapeutic target (30–33) and have the potential to become a novel approach in breast cancer treatment. However, it should be considered that although the RNAi strategy is effective and promising for gene-specific targeting and new RNAi delivery methods are under intensive investigation, efficient RNAi delivery methods are still lacking. Additionally, the phenomena of off-target RNAi is also commonly encountered in practice and any RNAi-based medicine must be carefully evaluated prior to its use in clinics.

## Figures and Tables

**Table I:** Expression of SNCG mRNA in MCF-7 cells (mean ± SD).

Transfected construct	SNCG mRNA concentration
SNCG-siRNA-1	0.624±0.010^a^
SNCG-siRNA-2	0.626±0.013^a^
SNCG-siRNA-3	0.634±0.008^a^
SNCG-siRNA-4	0.631±0.010^a^
Non-specific siRNA control	0.976±0.076^b^
Empty vector control	0.983±0.052^b^
F	147.42
P-value	<0.001

a P<0.05 vs. control;

b P>0.05 between the two controls. SNCG; synuclein-γ.

**Table II:** Expression of SNCG protein in MCF-7 cells (mean ± SD).

Transfected construct	SNCG protein concentration
SNCG-siRNA-1	4.27±0.12^a^
SNCG-siRNA-2	4.19±0.22^a^
SNCG-siRNA-3	4.15±0.14^a^
SNCG-siRNA-4	4.17±0.13^a^
Non-specific siRNA control	7.92±0.22^b^
Empty vector control	8.02±0.13^b^
F	481.06
P-value	<0.001

a P<0.05 vs. control;

b P>0.05 between the two controls. SNCG, synuclein-γ.

**Table III:** Effects of SNCG-siRNA suppression on clonogenicity in MCF-7 cells.

Group	Clonogenic rate (%)
Liposome control	32.33±2.52
Empty vector control	28.33±3.51
Non-specific siRNA control	32.00±4.36
SNCG-siRNA-3	18.67±1.53^a^
F	12.189
P-value	0.002

a P<0.05 vs. control. SNCG; synuclein-γ.

**Table IV:** Effects of SNCG-siRNA suppression in MCF-7 cells invasiveness assessed by Boyden chamber assays.

Group	Number of migrated cells
Liposome control	103.53±1.33
Empty vector control	100.93±1.81
Non-specific siRNA control	101.20±1.71
SNCG-siRNA-3	68.80±10.43^a^
F	572.108
P-value	<0.001

a P<0.05 vs. control. SNCG; synuclein-γ.
